# Investigation of the Utility of Multivariate Meta-Analysis Methods in Estimating the Summary Dose Response Curve

**DOI:** 10.34172/jrhs.2022.96

**Published:** 2022-12-29

**Authors:** Melepurakkal Sadanandan Deepthy, Kalesh Mappilakudy Karun, Kotten Thazhath Harichandrakumar, Narayanapillai Sreekumaran Nair

**Affiliations:** ^1^Department of Biostatistics, Jawaharlal Institute of Postgraduate Medical Education and Research, Puducherry, India; ^2^Division of Biostatistics, Malankara Orthodox Syrian Church Medical College, Kolenchery, Ernakulam, Kerala, India

**Keywords:** Dose-response meta-analysis, Multivariate meta-analysis, Parkinson’s disease, Restricted cubic spline model, Zonisamide

## Abstract

**Background:** Traditional meta-analyses often assess the effectiveness of different doses of the same intervention separately or examine the overall differences between intervention and placebo groups. The present study aimed to model the effect sizes obtained from different doses in multiple studies using a two-stage dose-response meta-analytic approach while taking dose variations into account.

**Methods:** Different dose-response meta-analysis models using linear, quadratic, and restricted cubic spline (RCS) functions were fitted. A two-stage approach utilizing multivariate meta-analysis was performed and the obtained results were compared with those of the univariate meta-analysis. A random effect dose-response meta-analysis was performed using data from an existing systematic review on combination therapy with zonisamide and anti-Parkinson drugs for Parkinson’s disease. The effective or optimum dose for producing maximum response was also investigated. Moreover, a sensitivity analysis was performed by changing the knots of the RCS model.

**Results:** Dose-response meta-analysis was performed using data from four double-blinded randomized controlled trials with 724 and 309 patients with Parkinson’s disease in dose and placebo arms, respectively. The quadratic model yielded the smallest Akaike information criterion (AIC), compared to the linear and RCS models, indicating it to be the best fit for the data.

**Conclusion:** Compared to the traditional approach, the two-stage approach could model the dose-dependent effect of zonisamide on the Unified Parkinson’s Disease Rating Scale (UPRDS) part III score and predict the outcome for different doses through a single analysis.

## Background

 The dose-response relationship is frequently investigated in clinical, epidemiological, and pharmacological studies.^1^ The effectiveness of any pharmacological intervention varies depending on the concentration of the drug administered, which makes it important to unveil and understand the dose-response relationship. Traditional meta-analyses often assess the effectiveness of different doses of the same intervention separately or examine the overall differences between intervention and placebo groups without consideration of the dose variations.^2^ Sometimes a subgroup analysis is also performed by classifying the doses as high and low which results in the loss of information about the intermediate doses.

 Dose-response or dose-effect relationship meta-analysis overcomes this limitation by modeling the relationship between effect sizes and different doses obtained from multiple studies.^3^ More importantly, it helps to incorporate the dependency between the study-specific effect estimates arising from the use of the same referent, compared to the conventional approach.^2^ It also helps researchers understand how the effect of an intervention varies as a function of its dose. There are two kinds of dose-response meta-analytic approaches; namely, one-stage and two-stage dose-response meta-analysis. They are used to study the shape of the dose-response association using aggregated data from multiple studies.^4^

 In the current study, a two-stage dose-response meta-analysis was performed using data from an existing systematic review on combination therapy with zonisamide and anti-Parkinson drugs for Parkinson’s disease.^5^ Parkinson’s disease is a type of central nervous system ailment that causes patients to lose their balance, induces difficulty in walking, and causes a lack of coordination. Various medications, such as levodopa, safinamide, dopamine agonists, and zonisamide are used to treat Parkinson’s disease.Among them,zonisamide is also used to treat epilepsy and psychiatric disorders.^6,7^

 Results of a few primary studies conducted in Japan have shown that zonisamide combination therapy improves motor and non-motor functions in patients with Parkinson’s disease.^8-10^ Although there have been few reviews and one meta-analysis about the effect of zonisamide on Parkinson’s disease, information on the dose-response relationship is not evident in these studies.^5,11-13^ The primary outcome evaluated in most of the studies was the unified Parkinson’s disease part III rating scale.^14^ It includes 14 items that examine speech, facial expression, finger and hand movements, posture, gait, and the effects of tremor, rigidity, and bradykinesia. Each item is scored from 0 to 4, where 0 indicates normal and 4 indicates severely affected. It is commonly used to assess the disease severity, progression, and response to treatment.

 Secondary outcomes, such as the Unified Parkinson’s Disease Rating Scale (UPDRS) part II score, total score, wearing off time, and adverse events were also studied. The pairwise meta-analysis available in the literature only investigated the overall improvement in UPDRS part III score between the zonisamide and placebo using all doses of zonisamide.^5^

 This study aimed to demonstrate the utility of dose-response meta-analysis with multivariate methods and compare the obtained summary results with those of a univariate meta-analysis. Dose-response meta-analysis will also help to predict the dose at which the maximum response is achieved, compared to the traditional meta-analysis. These reasons could make dose-response meta-analysis more clinically relevant than traditional meta-analysis. The reporting of dose- response meta-analysis can be done in accordance with the G-dose (43 items) checklist for reporting dose- response meta-analysis.^15^

## Methods

 Before the dose-response meta-analysis, estimates for different dose versus placebo comparisons were retrieved from individual studies. Pooled effect estimates were estimated for the different dose versus placebo comparisons. The data for this demonstration was taken from an existing systematic review on combination therapy with zonisamide and anti-Parkinson drugs for Parkinson’s disease.^5^

###  The Meta-Analytic Framework: Two-Stage Dose-Response Meta-Analysis

 A statistical model for modeling the dose-response relationship of correlated differences in mean scores of UPDRS part III with different dose variants was examined through a two-stage dose-response meta-analysis.^3^ In the first stage of the dose-response meta-analysis, once the effect estimates and their variance-covariance structure were obtained, a dose-response model was fitted in each study. The study-specific regression coefficients are often obtained by fitting appropriate linear or non-linear functions, such as Emax, quadratic, logistic, fractional polynomial, flexible piecewise linear, and restricted cubic spline (RCS). These were later combined using multivariate meta-analysis in the second stage of the study.^16-18^ The RCS and quadratic models have the added advantage of flexibility and can be performed if at least three levels of quantitative exposure exist (i.e., two different doses along with placebo). Study-specific trends were graphically plotted along with the study estimates and 95% confidence intervals (CIs). The linearity assumption was checked using Wald’s test, and a *P* value less than 0.05 was considered statistically significant.

 Once the vector of correlated effect estimates (*m*_i_) and the corresponding variance-covariance matrix (*S*_i_) was obtained from each study, the study-specific function could be defined as the following:


(1) $$m_{i}=f\left(z_{i},\beta_{i}\right)+\varepsilon_{i}$$

Where, $\varepsilon_{i}∼N\left(0,{\hat{\Omega }}_{i}\right),\;i=1,2,....n$ and ${\hat{\Omega }}_{i}$ is the covariance matrix.

 Linear, quadratic, and RCS models given below^3^ were fitted and the model that provided the lowest Akaike information criterion (AIC) was preferred.


(2) 1. Linear function:Emizi=β1izi

(3) 2. Quadratic function:Emizi=β1izi+β2izi2

(4) 3. The RCS model with three knots p1, p2 and p3:Emizi=β1iz1i+β2iz2i

Where *m*_i_ is the vector of correlated effect estimates, *z*_i_ is the vector of doses, *β*_1i_ and *β*_2i_ are regression coefficients, *z*_1_ and *z*_2_ are the two transformations


$$\begin{array}{l} z_{1}=z\\ z_{2}=\frac{{\left(z-p_{1}\right)}_{+}^{3}-\frac{p_{3}-p_{1}}{p_{3}-p_{2}}{\left(z-p_{2}\right)}_{+}^{3}+\frac{p_{2}-p_{1}}{p_{3}-p_{2}}{\left(z-p_{3}\right)}_{+}^{3}}{{\left(p_{3}-p_{1}\right)}^{2}} \end{array}$$

(Note: the notation “ + ”, with v_ + _= v if v ≥ 0 and v_ + _= 0 otherwise)

 The knots for the RCS model were specified at the 25^th^, 50^th^, and 75^th^ percentiles of the overall dose distribution. Generalized least square estimation was used to estimate the study-specific dose-response coefficients from *m*_i_, *S*_i_, and the design matrix *X*_i_ as follows:


$${\hat{\beta }}_{i}={\left(X_{i}^{T}S_{i}^{-1}X_{i}\right)}^{-1}\left(X_{i}^{T}S_{i}^{-1}m_{i}\right)\;and\;var\left({\hat{\beta }}_{i}\right)={\left(X_{i}^{T}S_{i}^{-1}X_{i}\right)}^{-1}$$

In the second stage, a multivariate meta-analysis was used to combine the vector of study-specific dose-response coefficients $\left(\hat{\beta }\right)$ and the variance-covariance structure acquired $\left({\hat{\Omega }}_{i}={\hat{\beta }}_{i}\right)$ from different studies. The vector of pooled coefficients and its variance covariance matrix are given below^19-21^:


$$\hat{\beta }={\left(\sum_{i=1}^{n}w_{i}^{*}\right)}^{-1}\left(\sum_{i=1}^{n}w_{i}^{*}{\hat{\beta }}_{i}\right)$$

$w_{i}^{*}={\left(\hat{T}+{\hat{\Omega }}_{i}\right)}^{-1}$ is the weight assigned to the m^th^ study. $\hat{T}$ is the between study variance covariance matrix, and ${\hat{\Omega }}_{i}$ is the within-study variance-covariance matrix.


$$Var(\hat{\beta })\approx {\left(\sum_{i=1}^{n}w_{i}^{*}\right)}^{-1}$$

The pooled dose-response curve using $\hat{\beta }$ and $Var(\hat{\beta })$ derived through multivariate meta-analysis for a set of z dose values was provided by Em^z=fZ,β^ along with an approximate 95% CI:


Em^z±Zα2diagfZ,β^TVarβ^fZ,β^

The results are presented as a graph using the predicted outcomes for a set of z dose values. Heterogeneity across study-specific trends was assessed using a multivariate extension of Cochran’s Q test for heterogeneity and quantified using I^2^. An I^2^ > 50% was considered to indicate considerable heterogeneity. The predicted responses using the models were compared to the observed pooled effect estimates for different doses versus placebo in the univariate meta-analysis. The dose at which the maximum observed response was determined. The effective doses required to produce 50% and 80% of the maximum response were also estimated. A sensitivity analysis was performed for the RCS model by specifying alternative knots at the 10^th^, 50^th^, and 90^th^ percentiles of the overall distribution of doses to determine how sensitive are the predicted pooled effect estimates to variations in the location of knots. All statistical analyses were carried out using *dosresmeta* package available in R software version 4.1.1. ^22,23^

## Results

 In the current study, four clinical trials that examined the dose-dependency relationship between zonisamide and UPDRS outcomes were used for analysis. The characteristics of the included trials are listed in Table 1. The sample sizes of the included studies were within the range of 136-375. zonisamide doses of 0, 25, 50, 100, and 200 mg/d have been used in different studies. The pooled difference between the mean scores of intervention and placebo for UPDRS part III were found to be 1.37 (95% CI: 0.31, 2.43), 2.27 (95% CI: 1.25, 3.29), and 2.67 (95% CI: 0.73, 4.61) for 25, 50, and 100 mg/d from three, four, and two studies, respectively. Only one study investigated a dosage of 200 mg/d, which reported a reduction in UDRS part III score of 5.60 (95% CI: 1.60, 9.60).

**Table 1:** Characteristics of the trials included and aggregate dose-response data from four randomized control trials investigating the effectiveness of zonisamide in patients with Parkinson’s disease

**Study ID/design**	**Sample size (n)**	**Dosage of zonisamide (mg)**	**UPDRS part III**	
			**Mean**	**SD**
AD810N-202-1				
Double blind RCT (10 weeks)	34	200	9.2	7.92
	36	100	6.5	8.16
	34	50	6.4	7.80
	32	Placebo	3.6	8.61
Murata 2007				
Double blind RCT 12 weeks)	82	100	4.6	7.24
	85	50	5.8	7.38
	77	25	6.3	7.02
	82	Placebo	2.0	7.24
Murata 2015				
Double blind RCT (12 weeks)	121	50	3.8	5.50
	125	25	2.0	5.59
	129	Placebo	2.3	5.68
Murata 2015 A				
Double blind RCT (12 weeks)	66	50	5.5	7.03
	64	25	5.9	7.03
	66	Placebo	2.9	7.14

UPDRS, Unified Parkinson’s Disease Rating Scale; SD, standard deviation.

###  Dose-response relationship within studies 

**Figure 1:**
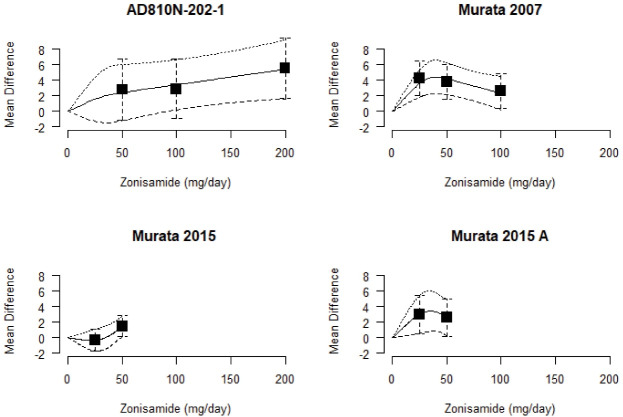
Study-specific difference in the mean scores of Unified Parkinson’s Disease Rating Scale part III for an increase in the dose of zonisamide. Zonisamide doses were modeled using the restricted cubic spline model. Black squares and whiskers represent the differences in mean UPDRS part III scores and their 95 % confidence intervals

 The study-specific predicted curves and CI (horizontal dashed lines), which were obtained using a RCS model, are shown in Figure 1. Squares with vertical lines represent the mean difference in UPDRS part III score along with 95% CI for different doses in each of the studies. The RCS model was fitted with three knots specified at the 25^th^, 50^th^, and 75^th^ percentiles (6.25, 37.50, and 50.0 mg/d) of the overall distribution of zonisamide doses.

###  Synthesis of coefficients obtained from multiple studies

 The coefficient ${\hat{\beta }}_{1}$ of linear model was estimated to be 0.025. The assumption of linearity was checked (*P* < 0.001) and evidence was found for the non-linear association between the zonisamide dosage and UPDRS part III score. The coefficients of the quadratic model ${\hat{\beta }}_{1}$ and ${\hat{\beta }}_{2}$ were estimated to be 0.065 and -0.0003. In the pooled dose-response meta-analysis model (RCS), the pooled difference in mean UPDRS part III motor score using 0 mg/d as a reference was 0.08z_1 _- 0.03z_2_. The corresponding 95% CI for a set of z dose of interest is given by the following:


$$\left(0.08z_{1}-0.03z_{2}\right)\pm 1.96\sqrt{0.0014z_{1}^{2}+0.0009z_{2}^{2}-0.002z_{1}z_{2}}$$

Heterogeneity across studies was found to be statistically significant (Q = 17.00, *P* = 0.01, I^2^ = 64.7%). The RCS model-based predicted differences in the mean score of UPDRS part III were 1.83 [95% CI: 0.21, 3.46] for 25 mg/d, 2.69 [95% CI: 1.21, 4.17] for 50 mg/d, 3.04 [95% CI: 1.69, 4.40] for 75 mg/d in comparison to placebo. The predicted differences in the mean score of UPDRS part III score using RCS, Linear, and quadratic models for different specified doses are given in Table 2. The RCS model indicated an increase in the difference in the UPDRS part III score with an increase in dose. Except for doses greater than 125 mg/d, the results were statistically significant for the RCS model, whereas the results were not significant from 125 mg/d for the quadratic model. The predicted differences using RCS and Quadratic models were close to each other for doses up to 125 mg/d. After that, the quadratic model showed a decreasing trend whereas the linear and RCS results showed an increasing trend and the results were closer to each other.

**Table 2:** Observed and Predicted differences in the mean scores of the Unified Parkinson’s Disease Rating Scale part III along with 95% confidence intervals using restricted cubic spline as well as Linear and quadratic models for specified doses

**Zonisamide** **Dose (mg/d)**	**Difference in mean UPDRS part III score (95% CI)**			
	**Observed **	**RCS **	**Linear **	**Quadratic **
25	1.37 (0.31, 2.43)	1.83 (0.21, 3.46)	0.63 (0.33, 0.93)	1.44 (0.31, 2.56)
50	2.27 (1.25, 3.29)	2.69 (1.21, 4.17)	1.25 (0.65, 1.85)	2.48 (1.11, 3.85)
75	No data	3.04 (1.69, 4.40)	1.88 (0.98, 2.78)	3.13 (1.69, 4.57)
100	2.67 (0.73, 4.61)	3.40 (1.03, 5.77)	2.50 (1.31, 3.70)	3.38 (0.40, 6.36)
125	No data	3.76 (0.08, 7.43)	3.13 (1.63, 4.63)	3.24 (-2.76, 9.24)
150	No data	4.11 (-0.93, 9.16)	3.76 (1.96, 5.55)	2.71 (-7.50, 12.91)
175	No data	4.47 (-1.98, 10.92)	4.38 (2.28, 6.48)	1.78 (-13.72, 17.29)
200	5.60 (1.60, 9.60)	4.83 (-3.04, 12.69)	5.01 (2.61, 7.41)	0.46 (-21.42, 22.34)
AIC	No data	-11.44	-12.74	-33.65
${\hat{ED}}_{50}$	No data	36.89	99.98	30.37
${\hat{ED}}_{80}$	No data	132.31	159.99	57.29

AIC, Akaike information criterion; UPDRS, Unified Parkinson’s Disease Rating Scale; RCS, restricted cubic spline.

 Among all three models, thequadratic model gave the smallest AIC = -33.65 in comparison to the other two models indicating it to be the best fit to the data. The RCS model gave an AIC = -11.44 similar to that of the linear model (AIC = -12.74). Even though a smaller AIC was obtained for the linear model, compared to the RCS model, the difference was negligible which may be due to the fewer number of dose categories in each study which may not be sufficient to detect a non-linear relationship.

**Figure 2:**
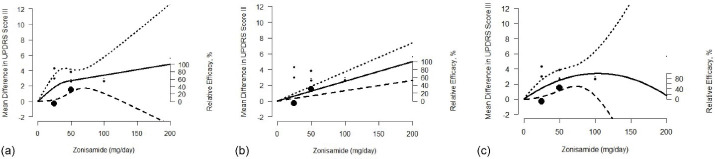
Pooled doses response association between zonisamide and difference in means of UPDRS part III score using (a) restricted cubic spline, (b) linear, and (c) quadratic models

 The predicted pooled dose-response curve along with the CIs and the difference in mean UPDRS part III scores for different models are shown in Figure 2. Zonisamide doses were modeled using RCSs as well as linear and quadratic functions, respectively (Figure 2a, 2b, and 2c). The solid line represents the pooled dose-response association between zonisamide and the difference in mean UPDRS outcome while the dashed lines represent the 95% CIs. It should be noted that the placebo group served as the reference group. The circles indicate the difference in the mean UPDRS part III scores obtained from the individual studies. The size of each circle is proportional to the precision of the estimate. The percentage of the maximum predicted effect or relative efficacy is shown on the right axis.

 The results showed a significant association between increasing doses of zonisamide and the difference in the mean UPDRS part III score with the maximum response of 4.83 and 5.01 observed at *x*_max_ = 200 mg/d using RCS and linear models. Under the RCS model, the dose required to produce 50% and 80% of maximum response were estimated to be ${\hat{ED}}_{50}=36.89\;mg/d$ and ${\hat{ED}}_{80}=132.31\;mg/d$ . The ${\hat{ED}}_{80}$ of the linear and quadratic model was estimated to be 99.98 mg/d and 30.37 mg/d. Similarly, estimated values of ${\hat{ED}}_{80}$ for the linear and quadratic model are found to be 159.99 mg/d and 57.29 mg/d, respectively.

###  Sensitivity analysis

 A sensitivity analysis was performed for the RCS model by specifying the knots at the 10^th^, 50^th^, and 90^th^ percentiles (0, 37.5, and 100.0 mg/d) of the overall distribution of the zonisamide doses. The pooled difference in means in UPDRS part III motor score using 0 mg/d as reference and the corresponding 95% CI for a set of z dose of interest was calculated using the following:


$$\left(0.064z_{1}-0.05z_{2}\right)\pm 1.96\sqrt{0.0007z_{1}^{2}+0.0036z_{2}^{2}-0.003z_{1}z_{2}}$$

The AIC of the RCS with alternative knots was found to be -4.09 which was higher than that of the RCS model indicating it to be less fit to the data than the RCS model with knots at 25^th^, 50^th^, and 75^th^ percentiles. The predicted pooled difference in means of UPDRS part III motor score using both RCS models were found to be similar, hence, less variation was observed in the shape of the curves.

## Discussion

 Dose-response meta-analysis helps to estimate more comprehensive and reliable high grade evidence. According to this study, the quadratic model provided a better fit to the data. In addition, this study was the first to investigate the potential dose-dependent effect of zonisamide combination therapy on the UPDRS part III score in Parkinson’s disease. The findings regarding the effect of zonisamide on UPDRS part III score were in accordance with those of a prior meta-analysis which also showed an improvement in the outcome.^5^

 Previous meta-analyses only performed a pairwise meta-analysis between the zonisamide and placebo groups. However, there are significant differences in the doses studied across different studies ranging from 0 to 200 mg/d. In addition, the differences between doses of zonisamide and placebo were not considered in the existing pairwise meta-analysis. The optimal dose for reduction in the UPDRS part III score also has not been determined. A previous meta-analysis indicated an overall reduction in the UPDRS part III score in the zonisamide group when compared to the placebo group (weighted mean difference: 2.56 [95% CI: 0.92, 4.20, *P* = 0.002]), whereas the current study was able to predict an improvement above this for all zonisamide doses from 45 mg/d onwards using RCS model. An improvement was observed for zonisamide doses above the threshold of 20 mg/d in the RCS model and the results were statistically significant up to 125 mg/d.

 Although the dose-response meta-analysis revealed an improvement in response to the increase in dose for linear and RCS, the quadratic model predicted initially increasing and later decreasing trends. When compared to the observed pooled responses for different doses versus placebo, the effect estimates were higher except for 200 mg for RCS and Quadratic. The CIs were wider in both RCS and quadratic models, compared to the linear model.

 The ${\hat{ED}}_{50}$ estimated using quadratic and RCS models were similar, whereas ${\hat{ED}}_{50}$ of linear was found to be three times higher, compared to the RCS and quadratic models. Estimated values of ${\hat{ED}}_{80}$ under linear and RCS were almost equal but were higher than that estimated under Quadratic. The present study determined the dose that yielded the maximum response. The maximum response or greatest reduction in the UPDRS part III score of the zonisamide and placebo was observed as 4.83 and 5.01, respectively, at *x*_max_ = 200 mg/d using RCS and linear models. In the sensitivity analysis, no substantial differences were observed in the results that were predicted using RCS models with varying knots. Since only a few studies were available, no subgroup analysis could be conducted in the present study. In addition, there were fewer dose categories in each of the studies to explore other non-linear models. Moreover, the generalisability of the results to other populations and the long-term efficacy of zonisamide cannot be determined since all the studies were carried out in the Japanese population with a short duration of 10-12 weeks.^5^

 Dose-response meta-analysis cannot be performed if dose-wise responses are not mentioned in individual studies. Moreover, studies reporting at least two non-referent doses along with a placebo should be available to proceed with a two-stage dose-response meta-analysis. A one-stage dose-response meta-analysis can accommodate studies with a single non-referent dose but will provide similar results to those of a two-stage analysis when studies with at least two or more non-referent doses only exist.

 The present research did not include any studies with a single comparison; therefore, a two-stage approach was employed. Since it has been made mandatory to register the studies in the scientific platform and provide the data in various public data depositories, the limitations mentioned above can be dealt with in future studies by obtaining the required dose-wise information from the raw data.

## Conclusion

 Compared to the traditional approach, dose-response meta-analysis could provide the effects of different doses of the same drug on the outcome through a single analysis. The quadratic model showed a better fit than other models with the lowest AIC value. Traditional meta-analysis has shown that zonisamide consumption results in an improvement in the UPDRS part III score. The current dose-response meta-analysis confirmed that combination therapies of zonisamide along with anti-Parkinson drug could result in improvement in the UPDRS part III score. The efficacy of different doses of zonisamide on the outcome was estimated; however, the selection of the appropriate dose also required the consideration of other outcomes, such as adverse events at various doses.

**Highlights**

- Traditional meta-analysis estimates either overall efficacy of a drug or for each dose separately.
- Dose-response meta-analysis models the dose-dependent effect of the intervention on the outcome.
- Efficacy of different doses of a drug on the outcome is estimated in a single analysis.
- A summary curve and heterogeneity across study-specific trends were estimated.
- Results of conventional and two-stage dose-response meta-analyses were compared.

## Acknowledgments

 The authors would like to thank Atul Goel, Senior Resident, Department of Neurology, JIPMER, Puducherry, India for helping with this project.

## Conflict of interest

 There is no potential conflict of interest.

## Ethical approval

 The study was carried out using the data from available literature; therefore, the Ethics committee granted a waiver of consent for the study.

## Funding

 This study was not funded.
